# An unusual presentation of neonatal rhabdomyosarcoma: a case report

**DOI:** 10.3389/fped.2023.1233334

**Published:** 2023-10-27

**Authors:** Danielle Strah, Kelly Stanley, Kelsie Oatmen, Ranjit I. Kylat, Megan Dishop, Michelina de la Maza

**Affiliations:** ^1^ Department of Pediatrics, Banner University Medical Center—University of Arizona, Tucson, AZ, United States; ^2^ Department of Pediatrics (Neonatology), Banner University Medical Center—University of Arizona, Tucson, AZ, United States; ^3^ Department of Pathology, Phoenix Children’s Hospital—University of Arizona, Phoenix, AZ, United States; ^4^ Department of Pediatrics (Hematology/Oncology), Banner University Medical Center—University of Arizona, Tucson, AZ, United States

**Keywords:** rhabdomyosarcoma, neonatal, case report, metastatic, congenital

## Abstract

A full-term infant with an unremarkable prenatal course presented at birth with a large midline facial mass and smaller masses in the head and neck. In addition, multiple diffuse flesh-colored nodules spread along all the upper and lower limbs. An extensive evaluation to cover a broad differential diagnosis of infectious, lymphatic/vascular, and oncologic etiology was undertaken. The initial suspicion was confirmed by biopsy of the skin lesion as congenital alveolar rhabdomyosarcoma (RMS). RMS is the most common soft tissue sarcoma that occurs in childhood. However, neonatal RMS is exceedingly rare. The infant’s initial treatment included vincristine, dactinomycin, and cyclophosphamide in addition to salvage ifosfamide and etoposide, which were dose-adjusted for age. Herein, we present a case of an infant with RMS who showed initial improvement before relapsing and succumbing to her disease at 5 months of age. A review of the limited literature available on this rare condition and newer treatment regimens with improved mortality rates is performed.

## 1. Introduction

A full-term infant was brought to our tertiary hospital for evaluation of a large facial mass, which was not detected prenatally. The mother received appropriate routine prenatal care with reportedly normal ultrasound scans. Upon the initial examination of the infant, multiple smaller masses in the head and neck region, as well as diffuse flesh-colored nodules covering all four extremities, were noted, in addition to the large midline mass. The initial differential diagnosis was broad and included infectious, lymphatic/vascular, and oncologic causes, prompting laboratory and imaging work-up, which led to a diagnosis of congenital alveolar rhabdomyosarcoma (RMS) confirmed by a biopsy review by pathology.

 RMS is a high-grade malignant neoplasm with a morphologic appearance that resembles a developing skeletal muscle (1, 2). It is the most common soft tissue sarcoma that occurs in childhood with an overall incidence of approximately 4.4 cases per 1 million individuals under 20 years old, representing about 3% of all pediatric cancers (1, 2). Neonatal RMS is an exceedingly rare presentation of pediatric RMS with cases of congenital RMS accounting for 1%–2% of all pediatric RMS cases (3). The most common primary site of neonatal RMS is the non-orbital, non-parameningeal head and neck regions, and the embryonal histologic subtype is more common than the alveolar subtype (4). Compared with older children, neonatal alveolar RMS is more often associated with multiple skin nodules and early brain metastases (3).

Herein, we describe a rare case of an infant with FOXO1 fusion-positive congenital alveolar RMS presenting with multiple cutaneous lesions. To the best of our knowledge, this is only the seventh reported case of congenital alveolar RMS presenting as diffuse multifocal cutaneous lesions, also described as a “blueberry muffin baby” in the literature (5–10). In addition, this case is unique as the infant presented with extensive non-cutaneous metastatic disease at birth, which has not been well described previously.

## 2. Case presentation

A full-term infant was admitted to our tertiary hospital for evaluation of masses. She was born to a 19-year-old gravida 1 with appropriate prenatal care. During the second trimester of pregnancy, the mother had elevated liver enzymes requiring hospitalization. During hospitalization, she was evaluated for infections, preeclampsia, cholecystitis, and hepatic injury, and magnetic resonance imaging (MRI) was also done. Her abdominal MRI was normal apart from biliary sludge. Her serology did not reveal an acute hepatic infection or evidence of an intrauterine infection, and her liver enzymes normalized in 1 week. Prenatal fetal ultrasound scans performed at routine intervals showed no abnormalities.

**Figure 1:**
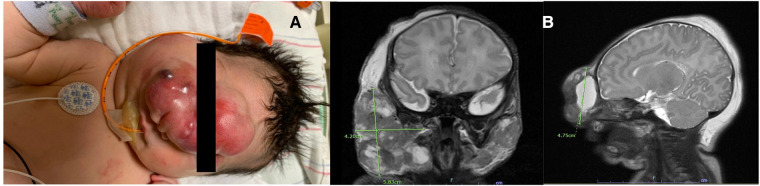
(**A**) Image of the infant's face taken on presentation demonstrating numerous facial lesions, particularly large midline face mass. The mass extended centrally from her forehead, down the length of her nose, to the midway on her right cheek, obscuring the right eye completely and the left eye partially. An additional large round mass was located above her right parotid gland with no extension noticed in her mouth, including the hard palate. (**B**) Initial MRI of the brain/face showing lesions predominately in the midline of the infant's face as well as the right parotid gland.

The infant was born via an uncomplicated spontaneous vaginal delivery with no requirement for resuscitation. Her initial physical exam was significant for a large midline mass distorting her eyes and nose with additional mass above her right parotid gland (Figure 1A). She also had multiple flesh-colored nodules with a slight blue hue spread on her bilateral upper and lower extremities, trunk, and back with sparing of her palms and soles. The initial differential diagnosis was broad and included infections, vascular/lymphatic malformations, and oncologic processes.

Imaging evaluation was initiated with an MRI of the brain and face, which showed three large mixed cystic and solid lesions on the mid-face and bilateral parotid regions. The mass lesions were primarily subcutaneous, along with deep tissue involvement and some mass effect of the right carotid space. No evidence of calvarial or intracranial involvement was reported (Figure 1B). A facial ultrasound demonstrated cystic components with low flow to the lesions. Evaluation of whole-body MRI revealed diffuse metastatic disease with involvement of the skin, muscles, and osseous cortices, a large pancreatic mass, inguinal lymphadenopathy, involvement of the right pelvic sidewall, and possible involvement of the thymus and both lower lobes of the lung and pleura. Laboratory evaluation was done consisting of complete blood count (CBC), complete metabolic panel (CMP), alpha-fetoprotein (AFP), beta-human chorionic gonadotropin (βHCG), and coagulation profile, which were all normal for her age, except for a mildly elevated aspartate aminotransferase (AST) level of 161 U/L, lactate dehydrogenase (LDH) of 1,308 U/L, and D-dimer, which was moderately elevated to 4,585 ng/ml. An infectious workup was negative for intrauterine and perinatally acquired infections such as congenital syphilis, and no microbiological growth was found on multiple blood cultures. She underwent evaluation for additional defects or abnormalities associated with midline lesions; an echocardiogram, abdominal ultrasound, and full ophthalmologic evaluation were performed, which were normal.

**Figure 2:**
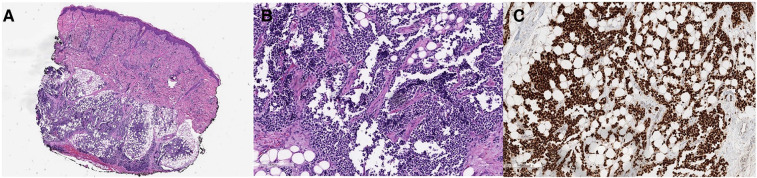
(**A–C**) A skin biopsy showed a dermal and subcutaneous infiltrate of hyperchromatic round cells (**A**, **H**&**E**). In some areas, the tumor cells line the thin fibrovascular septa and form aggregates within the intervening spaces, typical of the alveolar pattern of rhabdomyosarcoma (**B**, **H**&**E**). The diagnosis is confirmed by a strong nuclear positivity for myogenin (**C**, myogenin immunohistochemistry).

A biopsy of a right lower-extremity skin nodule showed a dermal and subcutaneous infiltrate of small round blue cells (Figure 2A). Focally, the tumor showed an alveolar pattern, indicated by some tumor cells lined along the fibrovascular septa and other discohesive tumors within intervening spaces (Figure 2B). The tumor cells showed cytoplasmic positivity for desmin and diffuse strong nuclear positivity for myogenin (Figure 2C). Molecular testing by fluorescence in situ hybridization (FISH) was positive for FOXO1, confirming the diagnosis of alveolar RMS.

**Figure 3:**
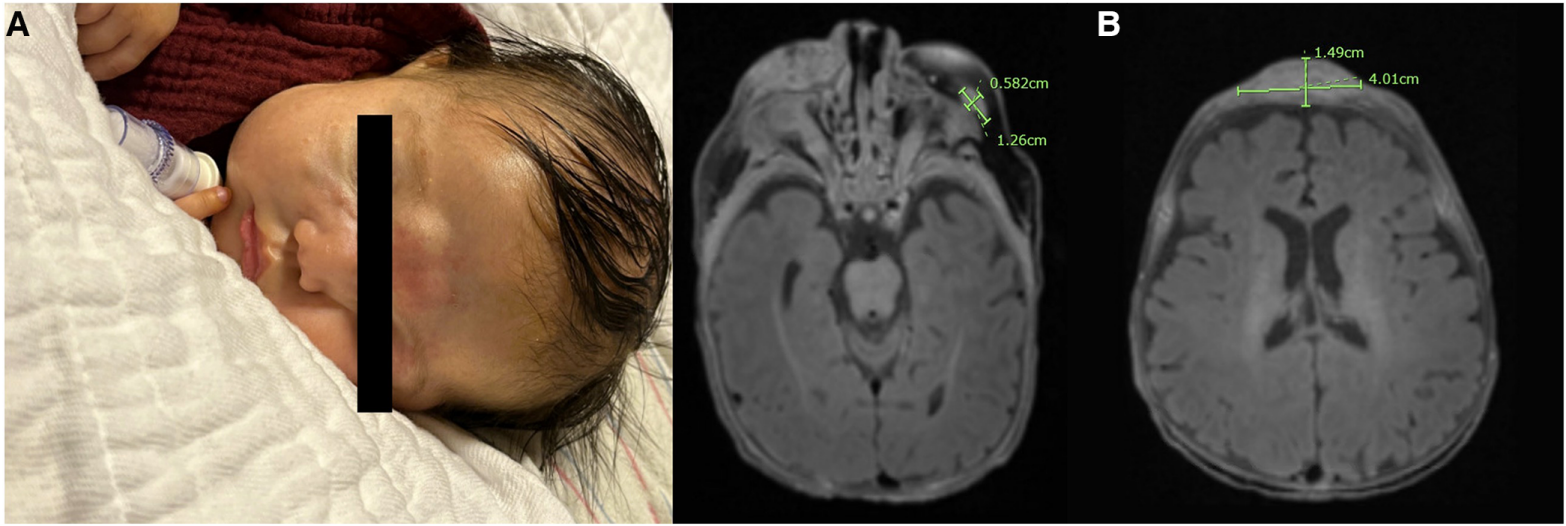
(**A**) Image of the infant's face taken after completion of cycle 1 with a notable reduction in the size of the facial lesions; the infant was able to fully open her eyes spontaneously at this point in therapy. (**B**) MRI of the brain taken prior to starting salvage therapy with an almost complete resolution of intracranial metastases and a notable reduction in the size of prior tumors.

She experienced early mass effects secondary to rapid progression of the facial lesion, including obstruction of the bilateral eyes, occlusion of the right nostril, superior vena cava (SVC) syndrome (secondary to compression from the masses during this phase of rapid growth), and stridor. She ultimately required a tracheostomy to secure her airway, given the concerns for possible mass compression. After extensive multidisciplinary meetings and counseling about all treatment options, the infant’s family decided to proceed with chemotherapy, which was started on day 20 of life. She received systemic chemotherapy with vincristine, dactinomycin, and cyclophosphamide at 50% weight-based dosing. Doses escalated with each cycle if tolerated. Her regimen was derived from the standard treatment for RMS as per the Children's Oncology Group with modifications made from previous case series of infants with RMS. Because of her tenuous nature, initial lumbar puncture, bone marrow biopsy, and PET scan were not performed. In addition, her staging would not have been affected by these results as she was already at stage IV at the time of diagnosis. Although surgery and radiation are typically performed in conjunction with chemotherapy, the multifocal nature of her lesions and the large size and vascularity of the facial lesion prohibited these steps for debulking. After the first cycle of chemotherapy, the lesions began to rapidly reduce in size, and she was able to open her eyes spontaneously (Figure 3A).

The patient tolerated dose escalation, achieving 100% of dosing by cycle 3. Post-cycle 3 imaging showed a decrease in the size of soft tissue lesions throughout her face, neck, and right orbit. However, numerous new enhancing lesions throughout the brain parenchyma and leptomeninges were concerning for brain metastases. Imaging of the chest, abdomen, and pelvis revealed a decreased size, number, and conspicuity of the diffuse metastatic disease. Lumbar puncture and bone marrow aspiration performed at this time were negative for the disease.

Her therapy was then transitioned to salvage treatment with 50% weight-based ifosfamide and etoposide, which was started in week 12 of life. She tolerated two cycles with continued improvement of all superficial lesions and an almost complete resolution of intracranial metastases (Figure 3B).

**Figure 4:**
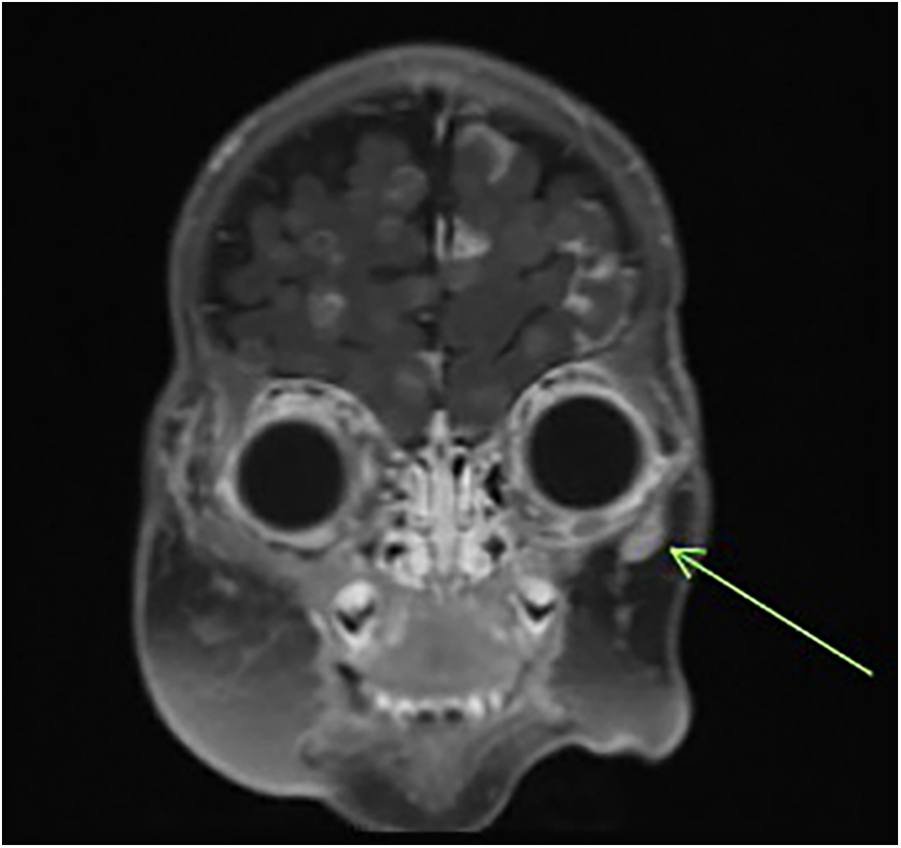
Follow-up MRI of the brain/orbit demonstrating diffuse intracranial metastatic spread and enlargement of facial lesions.

Post-salvage cycle 2, she was admitted for fever in the setting of neutropenia with a positive blood culture for *Klebsiella oxytoca*. Clinically, the facial lesions began to increase in size, and imaging confirmed diffuse disease progression with extensive intracranial metastases and an increase in the size of the midline nasal bridge/frontal scalp mass, right premolar soft tissue mass, and right anterior parotid mass, with development of internal necrotic changes (Figure 4). After a discussion with the multidisciplinary team, the family made the decision to discontinue the chemotherapy and transition to comfort care. She succumbed to her disease at 5 months of age.

## 3. Discussion

RMS is the most common soft tissue sarcoma that occurs in childhood. However, neonatal RMS is exceedingly rare (1–3). RMS that is diagnosed in the newborn period has a significantly worse prognosis than that of older children. The 5-year event-free survival (EFS) rate for infants diagnosed at <1 month is only 20%, and the 5-year overall survival rate is 40% (11). The four distinct histologic subtypes of RMS are embryonal, alveolar, pleomorphic, and spindle cell/sclerosing, with embryonal being the most common subtype in children (1, 12). The prognosis is increasingly worse for infants with alveolar RMS or metastatic disease (4, 13). The known favorable characteristics influencing survival outcomes include age at diagnosis, localized vs. metastatic, completeness of surgical resection, a tumor size of ≤5 cm, favorable tumor site, absence of lymph node involvement, confinement to the anatomic site of origin, and PAX7–FOXO1 fusion in FOXO1 fusion-positive RMS (14). The most important prognostic factor after metastatic status is FOXO1 status (15, 16). Treatment of neonatal RMS is further complicated by the high rates of chemotherapy toxicity; therefore, therapy is typically administered at lower, weight-based doses (3, 5, 12). Alveolar RMS can be further categorized as FOXO1 fusion-positive or fusion-negative, which is determined by the FOXO1 and PAX genes (1, 12). Pre-treatment staging utilizes the TNM system that includes the primary tumor site, size, invasion, nodal involvement, and distant metastasis (1, 3, 12). Risk stratification and treatment guidelines are further determined by the disease stage, the outcome of initial tumor resection, patient age, and fusion status (1, 3, 12). Treatment typically includes a combination of chemotherapy, surgery, and radiation (1–3, 12). Patients with FOXO1 fusion-negative tumors treated with ifosfamide, vincristine, dactinomycin, doxorubicin, ifosfamide, vincristine, actinomycin-D (IVA), maintenance vinorelbine, and cyclophosphamide showed a significantly better outcome than other strategies (17). Over the years, outcomes for localized disease have improved to 70%–80% relapse-free survival. However, progress has been tardy for metastatic and recurrent disease (1, 2). While metastatic disease is the most significant predictor of outcome, a worse prognosis is also associated with very young age (<1 year) and fusion-positive disease (1, 3, 5, 12).

To the best of our knowledge, our case is the seventh reported case of congenital alveolar RMS presenting as a “blueberry muffin baby,” but it also describes extensive non-cutaneous disease not described in other cases (5–10). The blueberry muffin lesions in the neonatal population lead to a consideration of not only prenatally acquired infections such as rubella, cytomegalovirus, coxsackie, and parvovirus B19 but also oncologic processes. The blueberry muffin rash can be a manifestation of dermal erythropoiesis or neoplastic infiltrations and has been described in neuroblastoma, RMS, Langerhans cell histiocytosis, and leukemia (18). The infant in the present case report had multiple poor prognostic factors such as metastatic disease at diagnosis, diagnosis at a very young age (<1 year), alveolar subtype, and fusion-positive disease (1, 3, 4, 11–13). Although she initially showed visible improvement with chemotherapy, the disease later progressed despite the patient reaching 100% dosing of initial therapy and completing two cycles of salvage therapy. Because of the extensive nature of her disease as well as the location of the large, midline, facial mass, this infant was not provided radiation therapy in an attempt to reduce her tumor size/burden, and also because of the vascular nature of the masses, she was unable to undergo a tumor debulking surgery. The infant ultimately succumbed to her disease. However, the patient likely had a longer life span than she would have had without therapy, with the treatment allowing for time outside of the hospital with her family.

This case highlights the importance of a broad differential for cutaneous lesions in infancy, the need for prompt tissue diagnoses, cooperation among a large subspecialty team, and family-centered care. This case also highlights the aspects of changing goals/treatment options to best align with the family's requirements for their children at different stages in the disease process. Future research should include further expansion of chemotherapy protocols and options for infants such as the one presented in this study, in whom radiation and surgery could not be performed.

Parental permission for this case review and for the publication of images was obtained.

## Data Availability

The original contributions presented in the study are included in the article/Supplementary Material, further inquiries can be directed to the corresponding author.
